# The availability of gonadotropin therapy from FDA-approved pharmacies for men with hypogonadism and infertility

**DOI:** 10.1093/sexmed/qfad004

**Published:** 2023-04-10

**Authors:** Benjamin J Borgert, Michael W Bacchus, Alexandra D Hernandez, Shelby N Potts, Kevin J Campbell

**Affiliations:** Florida State University College of Medicine, Tallahassee FL, United States; University of Florida College of Medicine, Department of Urology, Gainesville FL, United States; University of Florida College of Medicine, Department of Urology, Gainesville FL, United States; University of Florida College of Medicine, Department of Urology, Gainesville FL, United States; University of Florida College of Medicine, Department of Urology, Gainesville FL, United States

**Keywords:** compounding pharmacy, access to care, gonadotropin therapy, infertility, hypogonadism

## Abstract

**Background:**

Recent changes to the Biologics Price Competition and Innovation Act of 2009 have created barriers to accessing therapy for men utilizing gonadotropins for hypogonadism and infertility.

**Aim:**

In this study we sought to investigate ways to decrease disparities in the treatment of male hypogonadism by increasing access to gonadotropin therapy by identifying 503b outsourcing pharmacies which currently provide gonadotropin therapy.

**Methods:**

A review of 503b compounding pharmacies was performed using the online published registry available from the US Food and Drug Administration (FDA). Each pharmacy was contacted regarding their ability to provide gonadotropin therapy. Pharmacies were also queried regarding the impact of FDA-related legal changes and cost considerations.

**Outcomes:**

The study outcomes were the number and location of FDA-approved 503b compounding pharmacies supplying human chorionic gonadotrophin (hCG) and/or follicle-stimulating hormone (FSH) for the treatment of male hypogonadism and infertility.

**Results:**

The 81 503b-compounding pharmacies approved by the FDA to produce hCG and FSH therapy were identified using the FDA registry. Seventy-five of the 81 pharmacies responded to the survey (response rate 92.6%). Of the contacted pharmacies, 5 provided hCG (6.67%). Of the pharmacies offering compounded hCG, 4 offered FSH. No additional pharmacies offered compounded FSH. Eight pharmacies had previously provided hCG and FSH. Six of the 8 pharmacies that stopped making hCG and FSH cited the 2020 FDA mandate as the reason for halting compounding services. Of the 75 pharmacies that responded, only 1 pharmacy provided the cost for FSH ($287 per 100-IU vial), and 3 pharmacies provided the cost for hCG ($50-$83 per 10 000-IU vial).

**Clinical Implications:**

There are few FDA-approved outsourcing pharmacies currently providing male gonadotropin therapy, and increasing awareness of these pharmacies may decrease barriers to care for patients with male hypogonadism and infertility.

**Strengths and Limitations:**

The strengths of this article are the clinical utility of the data presented, as this article may serve as a tool for clinicians to increase patient access to therapy. All FDA-approved 503b outsourcing pharmacies were contacted, and 92.6% participated in this project. Limitations of this article were the following: no non-FDA–approved compounding pharmacies such as 503a pharmacies were contacted, participant-reported outcomes were utilized, and only 3 contacted outsourcing pharmacies provided a cost for FSH or hCG, allowing for an unknown degree of cost variability between outsourcing pharmacies.

**Conclusions:**

There currently exists limited access to FDA-approved compounded gonadotropin therapies for hypogonadism and male infertility, and these results demonstrate the barriers to hCG and FSH access and the need for additional treatment options for this vulnerable patient population.

## Introduction

Hypogonadism patients often present with low libido, erectile dysfunction, sleep disturbances, depression, and fatigue, among other symptoms.^1^ Prevalence of symptomatic hypogonadism (serum testosterone < 300 ng/dL) increases with age and affects 25%-39% of men aged 45 years and older.^1-3^ This population is subdivided into men who desire future fertility and those who do not.

Treatment for men desiring future fertility may involve the use of selective estrogen receptor modulators (SERMs), bioidentical hormones such as human chorionic gonadotropins (hCG), follicle stimulating hormone (FSH), and luteinizing hormone (LH), or aromatase inhibitors.^4^ No standard therapy or combination of therapies exists for treatment.^4^ Additionally, hCG is the only FDA-approved therapy for hypogonadism in men desiring future fertility.^4^

Therapeutic delivery mechanisms and how patients obtain the therapies vary substantially. SERMs and aromatase inhibitors are available through oral delivery mechanisms. In 1 study that compared costs of testosterone replacement to clomiphene citrate, clomiphene was given at 50 mg orally once daily.^5^ The average cost per month was US$83 dollars.^5^ Letrozole and anastrozole, commonly prescribed aromatase inhibitors taken orally at 2.5 mg daily or 1-5 mg daily, respectively, may cost less than 100 dollars per month.^6^ Clomiphene has been demonstrated to produce durable increases in testosterone. In a cohort of 86 patients treated with clomiphene 25 mg or 50 mg every other day, all patients had statistically significant increases in baseline testosterone at 19 months.^7^ Similarly, small studies have demonstrated aromatase inhibitor effectiveness in improving serum testosterone in individuals with testosterone concentrations <300 ng/dL and improving the testosterone/estradiol E_2_ ratio.^8^^,^^9^

Delivery and specific drug formulations become more complicated in FSH, LH, and hCG therapy.^10^^,^^14^ With the exception of 1 recombinant FSH (rFSH) formulation, FSH and LH are given as injections, frequently every other day, which may be challenging for some patients to administer and are less favorable as delivery mechanisms than oral medications.^10^ In FSH and LH therapy, there are basic distinctions between urinary-derived, highly purified (HP) FSH/HP LH, and rFSH and LH exists. Recombinant FSH is a biosimilar compound and is generally acquired as a trademarked drug.^10^ In the context of fertility, the efficacy of urinary-derived FSH/LH or recombinant biosimilars remain similar.^11^

Cost is also highly variable in FSH and +9 therapy.^12^^,^^13^ Cost varies by FSH formulation (HP-FSH vs rFSH) and by manufacturer. One study found FSH of all formulations to range from$360 to$1620 per month based on formulation and dosing (between 50 and 100 IUI per day).^12^ Whereas 1 pharmacy within our study reported the cost of urinary-derived FSH to be$287 per month at a dose of 100 IUI per day, costs for LH were similar in terms of price and variability compared to FSH.^13^

Similar to FSH and LH, hCG is given subcutaneously. For hCG therapy, regimens have been utilized such as dosing regimens including 2000 IU human chorionic gonadotropin (hCG) weekly, with patients receiving alternating biweekly and triweekly injections.^14^ Like FSH and LH, cost varies substantially, with 1 study reporting a monthly cost of$359 while pharmacies within our study reported monthly costs of less than$75 USD per month.^15^

**Table 1:** Questionnaire given to either a pharmacist or sales representative with knowledge of the products available from the given pharmacy.^a^’

**Standardized questionnaire for outsourcing pharmacies**
1. Does your pharmacy supply drug therapies that are used for the treatment of male infertility (specifically hCG, LH, or FSH)?
2. If no, have you ever offered this in the past?
3. If yes, why was this stopped and how long ago?
4. What, if any, interruptions or barriers have you encountered with supplying these medications?
5. What is your cost for hCG/FSH?

a This survey was presented to participating pharmacies in the context of increasing access to hypogonadal and male infertility pharmacotherapies for patients within our institution.

FSH, follicle-stimulating hormone; hCG, human chorionic gonadotrophin; LH, luteinizing horomone.

Given the high financial burden associated with these therapies and the lack of insurance coverage for nearly all of the therapies, physicians have had to advocate for patients both for insurance coverage and by identifying the lowest-cost providers of these medications.^16^ Recent changes to the Biologics Price Competition and Innovation (BPCI) Act of 2009, including the addition of FSH, LH, and hCG to the list of medications requiring a biological license in order to compound and deliver across state lines, and global supply chain shortages have created challenges for this population of patients.^17^ Herein, we discuss the availability of hCG, FSH, and LH for men seeking treatment for symptomatic hypogonadism and infertility, as well as the lack of hormonal therapy available from previously utilized 503b compounding pharmacies.

## Materials and methods

This study was deemed exempt from institutional review board need for approval. A review of the FDA-approved 503b compounding pharmacies was performed using the online published registry available from the FDA.^18^ A total of 81 FDA-approved 503b outsourcing pharmacies were identified as approved to compound hCG and FSH therapy. Each pharmacy was contacted primarily by phone and was asked a 5-question survey utilizing a standardized script, which is presented in Table 1. If available, the survey was presented to a pharmacy manager or other individual who had established experience with the services rendered at the respective pharmacy. Primary outcomes for this study were the number of 503B compounding pharmacies currently capable of offering compounded hCG, FSH, and LH and those currently offering compounded hCG, FSH, and LH.

Each pharmacy was surveyed to determine if they offered drug therapies directed toward male infertility. Positive responses were qualified by the type of medications offered. Pharmacies were also asked what drug therapies were previously offered for male infertility and the reason(s) for cessation. Subsequently, pharmacies were asked to note the presence of any interruptions or barriers to access that the pharmacy may have encountered when presently or previously supplying these medications. Additional questions regarding the impact of FDA-related law changes, supply chain interruptions, and cost considerations were also explored. Finally, the cost of hCG and FSH was requested. These standardized questions were sent via email to pharmacies that could not be reached by phone.

Response data were collected by each surveyor. Administration of the survey was conducted by the authors B.J.B and S.N.P. Descriptive statistical analyses were performed utilizing IBM SPSS software v26.^19^

## Results

**Figure 1:**
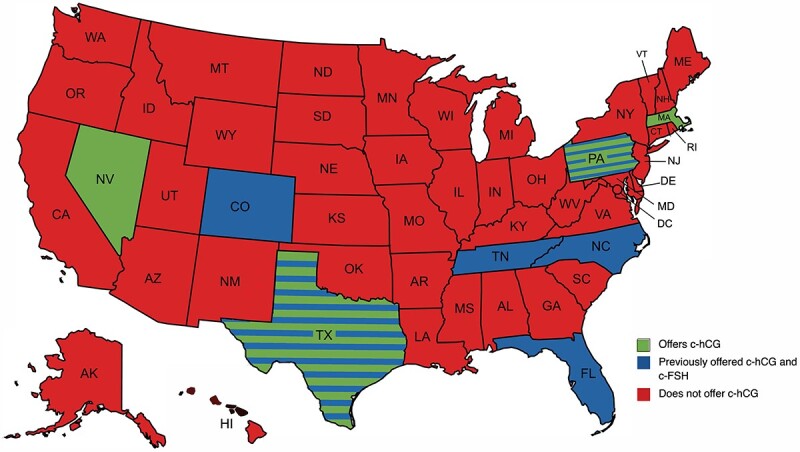
This map presents the locations of outsourcing pharmacies offering c-hCG and c-FSH. c-LH was not available from any of the survived pharmacies. States presented in green currently have outsourcing pharmacies which offer c-hCG and c-FSH. States presented in blue have outsourcing pharmacies which previously offered c-hCG and c-FSH but not longer do. States with blue and green stripes have outsourcing pharmacies which currently offer c-hCG and c-FSH as well as outsourcing pharmacies which previously offered c-hCG and c-FSH. States presented in red do not currently and have not ever had outsourcing pharmacies which offer c-hCG or c-FSH. c-FSH, compounded follicle-stimulating hormone; c-hCG, compounded human chorionic gonadotrophin; c-LH, compounded luteinizing horomone.

Eighty-one unique pharmacies were registered under FDA section 503b as outsourcing compounding pharmacies as of August 22, 2022. Seventy-five of the 81 pharmacies (92.6%) responded to inquiries made by investigators. Of the contacted pharmacies, 5/75 (6.67%) offered compounded hCG (c-hCG). Four of the 75 pharmacies (5.33%) offered compounded FSH (c-FSH). All pharmacies offering c-FSH also offered c-hCG. One pharmacy offering c-hCG did not offer c-FSH. None of the pharmacies that responded to this survey offered compounded LH. The percentages of pharmacies that responded to our survey, currently offer these agents, and had previously offered but had halted offering these agents are shown in Table 2. The geographic distribution of these pharmacies is shown in Figure 1.

**Table 2:** Descriptive statistics for outsourcing pharmacies listed under the FDA 503b registry for outsourcing pharmacies.

**Survey results from FDA-approved 503b pharmacies**		
	**Total**	**Percentage**
Outsourcing pharmacies	81	
Total response	75	92.60%
Currently provided		
FSH	4	5.33%
hCG	5	6.67%
LH	0	0.00%
Previously provided		
FSH	8	11.27%
hCG	8	11.43%
LH	0	0.00%
FDA mandate^*^	6	75.00%

^*^FDA Mandate refers to 503b Outsourcing pharmacies who stopped producing either hCG or FSH in response to the 2020 change of the BPCI Act of 2009. FSH, follicle-stimulating hormone; hCG, human chorionic gonadotrophin; LH, luteinizing horomone.

Secondary outcomes for this study were to determine the number of pharmacies that had halted production of c-hCG, c-FSH, and c-LH due to changes to the BPCI Act in March 2020, and the costs of c-hCG, c-FSH, and c-LH. Eight pharmacies had previously provided c-hCG and c-FSH (8/70) (11.43%). None of the pharmacies responding to this survey had previously offered LH. Six of the 8 pharmacies that stopped making hCG and FSH cited the 2020 FDA mandate as the reason for halting compounding services. Of the 75 pharmacies that responded, only 1 pharmacy was able to provide the cost for FSH ($287 per month), and 3 pharmacies were able to provide the cost for hCG ($50 to$83 per 10 000-IU vial).

## Discussion

For men seeking help with symptomatic hypogonadism and desiring future fertility, and men who were infertile due to hypogonadism of unknown etiology, limited therapeutic options are available. Among this population, the number of men with insurance coverage for symptomatic hypogonadism and hypogonadism–induced infertility represents an even smaller proportion of candidates. This population is similar to other populations requiring hormonal therapy, such as postmenopausal and infertile women. Given this paucity of therapeutic options, the 2009 BPCI Act created an abbreviated pathway for manufacturers of generic “biosimilars” to bring these products to market in hopes of increasing patient access to therapy and creating a more competitive, affordable market.^20^ This provision provided 2 options for patients: (1) patients could seek hormonal therapy from generic pharmaceutical manufacturers, and (2) patients could seek hormonal therapy from a compound pharmacy that prepared a particular dose or formulation for a particular patient by purchasing and preparing generic hormonal therapies.^17^

Clinicians and patients have utilized option 2 of the BPCI to obtain compounded gonadotropin therapies. Important distinctions between compounding pharmacies exist that have an impact on patient access to therapy. A basic description of these differences is presented in Table 3. Compounding pharmacies are subcategorized by which regulatory entity oversees their compounding practice.^21^ The two regulatory entities are state boards of pharmacy and the FDA. Compounding pharmacies regulated primarily by a state board of pharmacy are given the distinction of being a “503a compounding pharmacy.”^21^ These pharmacies must produce compounded products only on the basis of an individual prescription for an individual patient and generally cannot ship products across state lines.^21^ Compounding pharmacies given the distinction of a “503b compounding pharmacy” are regulated by the FDA.^21^ These pharmacies may or may not produce compounded products for individual prescriptions for individual patients.^21^ Additionally, 503b compounding pharmacies may ship products across state lines.^21^

**Table 3:** Basic description of key differences between 503a and 503b compounding pharmacies relevant to this study.^a^

**Basic comparison of 503a vs 503b compounding pharmacies**		
	**503a**	**503b**
Regulatory supervision	State Boards of Pharmacy	US Food and Drug Administration
Who do they compound for?	Individual prescriptions for individual Patients	Both individual prescriptions and large batches of product which can be distributed to healthcare facilities
Ship products across state lines?	No (<5% of sales must be across state lines)	Yes

a Several caveats and further key regulatory differences exist but are outside the scope of this discussion.

Historically, pharmaceutical compounding was essential for creating drug preparations which were not commercially available.^20^ These included liquid forms of medications for children, patients with dysphagia, and patients with allergies to dyes or other products in a manufactured medication.^22^ Total parenteral nutrition formulations and “magic mouthwash” are commonly utilized examples of compounding at work in our healthcare system.^22^ Compounding has evolved over time, and compounding pharmacies have expanded to meet the needs of whole populations of patients rather than individuals. Large compounding pharmacies today are referred to as “outsourcing pharmacies” and ship products across state lines. These pharmacies are given the distinction of being a 503b compounding pharmacy. Following the 2013 Compounding Quality Act (CQA), these “outsourcing pharmacies” operate under FDA legislation and must submit to review of sterilization practices and track product distribution.^20^^,^^22^^,^^23^

Outsourcing pharmacies are of interest because both physicians and patients frequently view compounded bioidentical/biosimilar pharmaceuticals as having lower costs than manufactured therapies.^24-27^ Other authors have previously reported that for FSH specifically, GoodRx prices for B-FSH per unit were$2.20, while a compounding pharmacy provided B-FSH for$0.20 per unit.^27^ In our research, the same outsourcing pharmacy was contacted and provided a similar price for FSH. Current FSH GoodRx prices are between$2.75 and$3.00 per IU.^28^

For hCG our research demonstrated that outsourcing pharmacies were able to provide hCG at a cost of between$50.00 and$88.33 per 10 000-IU vial, compared to a national GoodRx search, which yielded the lowest cost for generic hCG of$247.65 per 10 000 IU vial.^29^ A cost comparison between compounded and manufactured gonadotropin therapy is presented in Table 4. While our results confirm the work of prior authors who have reported that outsourcing pharmacies are often able to provide lower-cost gonadotropin therapies to infertile and symptomatically hypogonadal men, these pharmacies also underlie the general barriers men face with accessing affordable gonadotropin therapies.^16^ Prior to March 2020, only 13 outsourcing pharmacies nationally have provided compounded gonadotropin therapies for patients. Since that time, 61.5% of those pharmacies have halted compounding operations. Seventy-five percent of those halting production cited the March 2020 provision to the BPCI Act of 2009. The March 2020 provision added hCG, FSH, and LH, among other drugs, to a list of medications known as “biologics.” Once hCG, FSH, and LH are classified as biologics, they cannot be compounded unless the pharmacist has a Biologics License Application (BLA), unless a special exception was granted.^17^^,^^30^^,^^31^ A BLA requires an annual fee paid by outsourcing pharmacies to allow these pharmacies to produce and distribute biologic products across state lines. The annual cost of this application was$5998 in 2022.^32^ This fee coupled with the non–small business establishment fee ($18 999) and the reinspection fee ($17 472) for outsourcing facilities disincentivize outsourcing pharmacies from providing gonadotropin therapy to patients.^33^

**Table 4:** Costs of male hormone therapy from various distributors.

**Cost analysis of male hormone therapy**			
	Good-Rx^a^	Outsourcing pharmacy^b^	Brand name^c^
hCG (10 000 IU)	$247.65	$69.17	$357 (Pregnyl)
FSH (300 IU)	$861.17	$60.00	$1046 (Follistim aq)
LH	Not offered	None provided	Not offered

FSH, follicle-stimulating hormone; hCG, human chorionic gonadotrophin; LH, luteinizing horomone.

a The Good-Rx prices reflect the lowest prices from a national search of the brand listed in “brand name” column.

b The brand name column reflects the standard market price according to Good-Rx.

c The outsourcing pharmacy column reflects the average cost of gonadotropin therapy based on our survey results.

Despite the financial benefits associated with utilizing compounded pharmaceuticals, the safety and efficacy of these products have been an area of contention. Compounding pharmacies are not subjected to the same safety and quality testing as manufactured pharmaceuticals. Concerns have been raised about producing pharmaceuticals without such oversight. The gravest example of this lack of oversight occurred in 2012, when 76 individuals died from fungal meningitis traced to products produced by the New England Compounding Company.^34^ The Compounding Quality Act passed in 2013 attempted to increase oversight of 503b compounding pharmacies to prevent such contamination in the future.^23^ Despite this act, a report by the National Academies of Science, Engineering, and Medicine (NASEM) in 2020 entitled “The Clinical Utility of Compounded Bioidentical Hormone Therapy: A Review of Safety, Effectiveness, and Use” called into question the quality and safety of compounded biological hormone therapy (cBHT).^35^ In this review NASEM indicated “there is a dearth of high-quality research with a primary or secondary endpoint focused of the safety, effectiveness, and performance of cBHT preparations.”^35^ There is a repository of both positive and negative anecdotal evidence regarding the quality and safety of cBHT; however, an evaluation of these parameters is beyond the scope of this study. Clearly, a balance between availability of cBHT and the safety and quality of these products remains an area of both governmental and academic interest.

Despite highlighting challenges in mens’ access to compounded gonadotropin therapy, our study is not without limitations. First, while our overall response rate was 92.6%, the ability for those pharmacies offering compounded gonadotropin therapy to provide pricing for this therapy was limited. Sixty percent of pharmacies offering hCG were able to provide a cost per 10 000-IU vial. Additionally, only 1 pharmacy offering FSH was able to provide a price for the medication. This study utilized standardized survey methods and is therefore subject to intersurveyor variability. Additionally, as both pharmacists and pharmaceutical representatives were classified as qualified to answer questions regarding the availability of compounded gonadotropin, it is possible errors were reported by those surveyed. However, these experiences may increase the generalizability of our results as physicians or patients contacting approved “outsourcing pharmacies” may face similar challenges. Finally, it was not deemed viable to contact all compounding pharmacies nationally, and those who operate under a state board of pharmacy jurisdiction (503a compounding pharmacies) rather than FDA regulations may provide compounded gonadotropin therapy. These circumstances underlie the need for provider experience within their own practice environment to understand all options available for patients seeking gonadotropin therapy.

While our research demonstrates the barriers symptomatically hypogonadal and infertile men face with acquiring gonadotropin therapy, we commend the efforts of national regulatory bodies to ensure the safety and quality of compounded gonadotropin therapy through increased regulation. Additionally, we recognize the historic harms caused by compounding pharmacies, which necessitate these regulations. It is our hope that this research furthers the discussion surrounding patient access to gonadotropin therapy and improves the equity of mens’ hormonal healthcare.

## Conclusion

Biologics, such as hCG and FSH, have been demonstrated to provide therapeutic benefit in hypogonadal men seeking treatment while maintaining fertility. Furthermore, FDA-approved 503b compounding pharmacies serve an important role in providing these medications to this vulnerable patient population. In this work, we demonstrate the current availability of these medications and explore limitations and restrictions to widespread access of these agents. Further research is needed to better understand these limitations and explore new solutions to improve access to care.

## Funding

None declared.

*Conflicts of interest:* None declared.
